# Solute displacement in the aqueous phase of water–NaCl–organic ternary mixtures relevant to solvent-driven water treatment

**DOI:** 10.1039/d0ra06361d

**Published:** 2020-08-10

**Authors:** Joshua S. McNally, Zi Hao Foo, Akshay Deshmukh, Christopher J. Orme, John H. Lienhard, Aaron D. Wilson

**Affiliations:** Idaho National Laboratory P.O. Box 1625 MS 2208 Idaho Falls ID 83415-2208 USA aaron.wilson@inl.gov; Rohsenow Kendall Heat Transfer Laboratory, Department of Mechanical Engineering, Massachusetts Institute of Technology 77 Massachusetts Avenue Cambridge MA 02139-4307 USA

## Abstract

Twelve water miscible organic solvents (MOS): acetone, tetrahydrofuran, isopropanol, acetonitrile, dimethyl sulfoxide, 1,4-dioxane, dimethylacetamide, *N*-methyl-2-pyrrolidone, trifluoroethanol, isopropylamine, dimethylformamide, and dimethyl ether (DME) were used to produce ternary mixtures of water–NaCl–MOS relevant to MOS-driven fractional precipitation. The aqueous-phase composition of the ternary mixture at liquid–liquid equilibrium and liquid–solid endpoint was established through quantitative nuclear magnetic resonance and mass balance. The results highlight the importance of considering the hydrated concentrations of salts and suggest that at high salt concentrations and low MOS concentration, the salt concentration is governed by competition between the salt ions and MOS molecules. Under these conditions a LS phase boundary is established, over which one mole of salt is replaced by one mole of MOS (solute displacement). At higher MOS concentrations, MOS with higher water affinity deviate from the one-to-one solute exchange but maintain a LS boundary with a homogenous liquid phase, while MOS with lower water affinity form a liquid–liquid phase boundary. DME is found to function effectively as an MOS for fractional precipitation, precipitating 97.7% of the CaSO_4_ from a saturated solution, a challenging scalant. DME-driven water softening recycles the DME within the system improving the atom-efficiency over existing seawater desalination pretreatments by avoiding chemical consumption.

## Abstract 2

Water–NaCl–organic ternary mixtures evaluated with hydrates salt concentrations to reveal general phenomenon of the one-for-one molar displacement of NaCl by organic solutes as well as implications on solvent driven water treatments.

## Graphical abstract

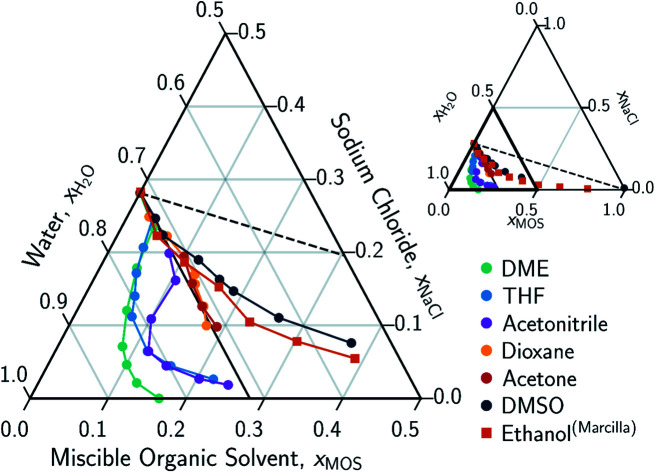

## 1. Introduction

**Fig. 1:**
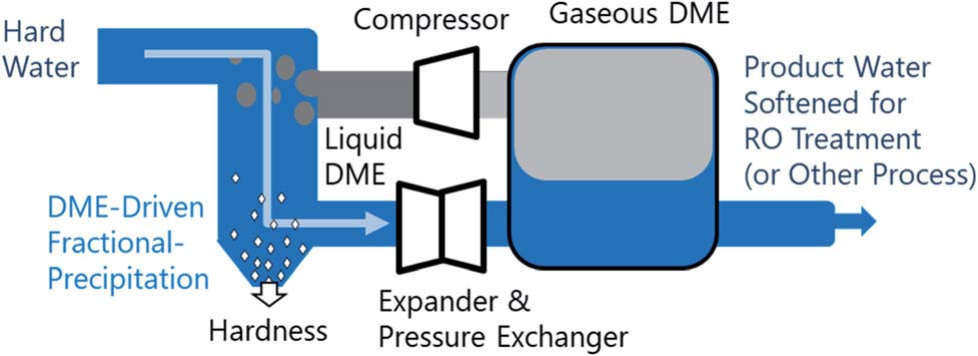
Conceptual design of dimethyl ether-based miscible organic solvent-driven fractional precipitation (FP). Hard water is mixed with liquified dimethyl ether (DME), driving the fractional precipitation of solutes from the saline brine solution. Solid precipitates are separated before the water–DME mixture is decomposed by decompression in an expander or the solution is heated to drive the vaporization of DME, which has a vapor pressure of approximately 5 bar temperature of 293 K. Gaseous DME then undergoes compression and liquefaction before being recycled, while the softened product water is passed to the next stage in the water treatment process. A pressure exchanger can be used to recover energy from the expansion process.

Fractional crystallization/precipitation (FP) occurs when a salt is selectively precipitated from a solution, at a concentration often below what would be predicted by its solubility product, through the manipulation of solution temperature, pressure, or composition. A water miscible or partially-miscible organic solvent (MOS) can be added to brine to induce a compositional change, selectively precipitating solutes from the mixture.^1–10^ Industrially, this process has been used for the energy-efficient bulk production of salt from the brine solutions.^11–16^ MOS-driven FP may conceivably augment current water treatment systems, improving water softening and driving the reduction of bulk total dissolved solutes (TDS). However, the removal of MOS from the low-TDS product water may lead to increased cost. Thus far, the difficulty of MOS removal has limited FP to applications suitable for salt production, where both the aqueous and organic phases are recycled within the process.^8,17–19^ To address the limitation imposed by residual solvent on the broader application of FP, this study explores the potential of a polar aprotic MOS, dimethyl ether (DME), that is biased for separation from water and can be efficiently removed from water, brines, and solids, as illustrated in Fig. 1. To understand the relative performance of DME, we characterized the aqueous phase of the liquid–liquid equilibria of twelve water–NaCl–MOS ternary mixtures, and compared the results to existing data available in the literature.^20–22^ The measurements presented provide key insights into the mechanism of MOS-driven FP, which have implications for fundamental electrolyte solvation phenomena.

DME exists in the gaseous phase at near ambient temperature and pressure (293 K and 1 bar). Elevated pressures (5 bar or more) are required to liquefy DME to an appreciable concentration in aqueous solutions. DME removal is easily accomplished through depressurization or heating, which is expected to offset some of the costs associated with operating a pressurized MOS-driven FP system. The avoidance of fugitive losses of MOS in product water and the resultant concentrates (both brines or solids) expands the operational envelope of MOS-driven FP towards a range of new applications, including pre-treatment for seawater desalination, general water softening, zero liquid discharge (ZLD), and hydrometallurgical purification. In addition, an effective MOS-driven FP process can potentially be adapted to function as a biocide, sterilization the water^23–26^ as well as a method to extract biological materials.

In the case of seawater reverse osmosis (SWRO), nearly half of the process footprint is dedicated to pretreatment. These pretreatments remove particulate, scalants, and biofoulants. The incumbent method of addressing biofouling is generally through the addition of biocides and chemical oxidation agents. These oxidation agents often need to be neutralized by more additives to reduce damage to the RO membrane's polyamide selective layer. Scalants, notably CaSO_4_, are controlled with the addition of anti-scalant chemicals.^27,28^ All of these chemicals are concentrated and discharged with the brine waste. The total impact of concentrating these chemicals into a brine discharge, like many potential environmental hazards, is not yet fully understood and would likely be best avoided. In addition to discharging hazards the consumptive use of chemical is not essential to the desired transformation and thus represents poor atom economy which includes the embedded energy and environmental impacts of producing those chemicals. By improving the atom economy and avoiding chemical discharge, DME-based SWRO pretreatment could be a green improvement over the incumbent technology.

If DME is to be applied in a widely-used industrial process, such as SWRO and other potable water treatment, the potential challenges around storage, transportation, handling, and toxicity must be understood. DME, in both its liquid and gaseous phases, is relatively well suited for use in water treatment due to a unique combination of chemical properties, including: (a) high polarity and ability to both absorb water and dissolve into water;^29^ (b) a well characterized vapor-compression cycle given its use as a refrigerant (R-E170);^30^ (c) low toxicity with current uses including food production and pharmaceutical systems;^31^ (d) low reactivity and not peroxide forming;^32^ and (e) low-cost as a mass-produced chemical that has potential applications as a fuel.^33^ In addition, DME has been explored as a drying reagent due to its ability to dewater porous materials. DME has yet to be applied in the fractional precipitation of salts from aqueous brines and slurries.^34^ A key benefit of using DME-based MOS-driven FP water softening is the high volatility of DME, which allows its complete removal from products.

The development of DME into an effective MOS for FP requires a thorough understanding of how an MOS induces the precipitation of ionic salts. If an optimal MOS is utilized, larger quantities of salt might possibly be removed per volume of MOS added to water. However, the mechanistic basis of this aspiration is not established. A variety of theoretical models have been proposed to explain MOS-driven FP, but currently lack extensive experimental validation. An “implicit-solvent” solution model suggests that the MOS may change the solution's dielectric properties, inducing a change in solubility characteristics. Under an “explicit-solvent” solution model, the MOS may act as a solute in the aqueous solution, thereby competing with the dissolved salt for explicit interactions with water. These non-idealities may also be examined through activity coefficient models that regress the underlying solvation mechanisms into several energy-based interaction parameters. Liquid–liquid equilibria are often modeled using the non-random two-liquid (NRTL) or universal quasichemical (UNIQUAC) activity coefficient models, with further adjustments, such as the incorporation of a Debye–Hückel term, to account for the impact of electrolytes.^35–39^ Some empirical models have also been developed with FP research, and these have their own complications.

The MOS-driven FP literature has not always distinguished between single-liquid-phase and two-liquid-phase systems that result from mixing brines and MOSs. Even when reports acknowledged the formation of two-liquid-phases in FP, the composition of those both phases is rarely recorded.^4^ When an MOS is added to a brine and precipitation occurs, without the formation of a second organic phase, the process is clearly an FP process. If more MOS is added and an organic phase forms,^4^ additional processes occur that cannot be described by an FP mechanism alone. The organic phase will selectively extract water from a brine inducing further precipitation of a salt. This water-selective extraction^40–59^ is a mechanism distinct from FP. Several conceptual and mathematical models have been developed to describe FP, but those models ignore these distinct mechanisms, thereby oversimplifying the process. Models that assume that MOS-driven fractional precipitation emerges from a single phenomenon rather than two different phenomena are not conceptually suitable for the optimization of MOS-driven FP processes.

Given the limitations of the data and models for MOS-driven FP, we instead look to the fundamental literature on the phase behavior of ternary water–salt–MOS mixtures. Surprisingly, very few experimentally determined phase diagrams exist for water–salt–MOS systems.^20–22,60–66^ To close the knowledge gaps associated with ternary phase behavior of water–NaCl–MOS systems for FP, we have experimentally obtained the aqueous phase boundaries for twelve different MOSs, expanding on four relevant datasets that we found in the literature.^20–22^ Based on these data, we have developed a conceptual model for the mechanism of MOS-driven FP.

## 2. Experimental

### 2.1. General

Nuclear magnetic resonance (NMR) spectra were acquired on a Bruker Avance III 600 MHz spectrometer with a magnetic field strength of 14.093 T, corresponding to operating frequencies of 600.13 MHz (^1^H). All NMR except the DME experiment were captured with a co-axial insert containing D_2_O (Cambridge Isotopes Laboratories). The ^1^H NMR spectra were collected with a 90° pulse and with 30 s to 60 s delays between scans. The T1 of every integrated shift was verified and most T1 relaxations were well under 2 s, and none were above 4 s. Calcium concentrations were measured with inductively coupled plasma optical emission spectrometry (ICP-OES), at a detection limit of 0.011 μg mL^−1^ Ca.

ACS grade NaCl, free of anticaking agent, was used after spending at least 48 hours in a vacuum oven at 150 °C. Solvents were obtained as anhydrous when possible. The MOS used in these studies included dimethyl ether (DME), acetone, tetrahydrofuran (THF), isopropanol (IPA), acetonitrile (MeCN), dimethyl sulfoxide (DMSO), 1,4-dioxane, dimethylacetamide (DMAc), *N*-Methyl-2-pyrrolidone (NMP), trifluoroethanol (TFE), isopropylamine (IPamine), and dimethylformamide (DMF). Acetone was dried for 30 min over 3 Å sieves. Nuclear magnetic resonance (NMR) established that the water to MOS mole ratio was less than 0.001 for all the MOS used in this study.

### 2.2. Aqueous phase studies of water–NaCl–MOS

Stock solutions of known masses of NaCl and distilled H_2_O were prepared. An MOS was added to 1–3 g of a stock NaCl solution. When the solution became cloudy, additions were slowed until a thin organic layer was clearly visible upon settling. After settling, 0.4 mL of the heavier aqueous phase was transferred to an NMR tube fitted with a coaxial insert containing D_2_O. T1 experiments were conducted to establish the relaxation time of water and the MOS. Quantitative NMR was conducted on the sample using 90 degree pulses, with delays (30–60 s) at least five times longer than the longest T1, with the temperature being regulated at 298 K throughout. NMR analysis allowed for the mole ratio of H_2_O to MOS to be established, while mass balance established the mole ratio of water to NaCl.

### 2.3. Aqueous phase studies of water–NaCl–DME

Solutions of known masses of H_2_O, D_2_O, and NaCl were produced, and 0.4 mL transferred to a Teflon NMR tube. The solution was frozen in liquid nitrogen. DME was condensed into the chilled NMR tube, and the tube was then capped. The Teflon tube containing the sample was allowed to warm to ambient temperature and self-pressurize. Provided an organic layer of DME was observed above the measurement region, the sample was then analyzed using NMR. If a DME layer was not observed, the sample was refrozen, and more DME was added. T1 experiments were conducted to establish the relaxation time of water and DME. Quantitative NMR was again conducted on the sample using 90° pulses, with delays (30–60 s) at least five times longer than the longest T1, with the temperature being regulated at 298 K. NMR analysis allowed for the mole ratio of H_2_O to MOS to be established, while mass balance established the mole ratio of H_2_O, and D_2_O, to NaCl.

### 2.4. Removal of calcium sulfate

Approximately 150 mL of saturated CaSO_4_ brine were pumped into a vessel consisting of a 50 mm glass tube with 5 mm glass walls and capped with threaded Teflon plugs and contained within a polycarbonate jacket. The plugs featured three taped bores to allow lines and pressure relief valves to be introduced from the top and the bottom of the vessel. Liquid DME was added to the system until a thin layer of organic appeared above the aqueous phase. The aqueous solution was mixed *via* recirculation using a gear pump for ∼5 min with the entrained solids then allowed to settle for ∼10 min. About 30 mL of the aqueous supernate were pumped using a gear pump through a 1.5 μm Nylon syringe filter into ambient atmosphere. The initial and final concentration of the solution were measured ICP-OES. The final concentration for CaSO_4_ was 14.5 μg mL^−1^ by Ca for a 97.7% removal from the saturated CaSO_4_ brine (626 μg mL^−1^ by Ca).

## 3. Results and discussion

### 3.1. Aqueous phase composition of water–NaCl–MOS ternary systems

A variety of MOSs with distinctly different solubility chemistries (fully water-miscible, partially water-miscible, polar protic, and polar aprotic solvents) were evaluated to determine the relative performance of DME for selective FP in ternary water–salt–MOS systems. Working with NaCl, a highly soluble salt, allows for the identification of gross trends and upper limits of the ternary water–salt–MOS systems as well as establishing a benchmark for water–salt–DME systems. Exploring the water–salt–DME composition space (DME being the working fluid in Fig. 1) using sparingly soluble salts (*e.g.*, CaCO_3_, MgCO_3_, CaSO_4_) is experimentally infeasible due to the difficulty in quantitatively adding DME and extracting controlled samples to measure the associated salt concentrations.

These ternary mixtures of known composition were produced from aqueous brines of known mass balance. Much like a titration to an endpoint, an MOS can be slowly added to the brine with agitation until either an organic phase separates from the mixture, or until the solid salt precipitates. The mole ratio of MOS to water of the saturated aqueous phase can be determined with quantitative ^1^H nuclear magnetic resonance (NMR) spectroscopy. With the mole ratio of NaCl : H_2_O and H_2_O : MOS established, the aqueous phase composition of ternary water–NaCl–MOS mixtures under saturated conditions can be calculated.

The compositions explored in this study focused on highly concentrated NaCl conditions where there was a very clear delineation in phase that was easily observed (generally 7 wt% NaCl or higher). This concentration range distinguishes this study from previous work on Setschenow constants which considered the “salting-out” of solvents with most of the work conducted at seawater concentrations (∼3.5 wt% NaCl) or less.^67–69^ This study is also distinct from studies concerning the Hofmeister series, which was developed to understand the salting-out of proteins by different salts (since extended to the salting-out of other materials). Most of the work with the Hofmeister series has addressed how different salts affect the solubility and partitioning (aqueous *versus* organic) of biological solutes.^70^ Hofmeister series studies generally do not systematically address the influence of concentration.^71,72^ To the best of our knowledge there has not been a compositional Hofmeister series study involving any of the ternary water–NaCl–MOS mixtures explored in this study.

Within this study once the relative concentrations of water–NaCl–MOS are determined, the concentrations of the species are expressed in terms of their effective mole fractions. Concentrated solutions are best described using mole fractions, which are unaffected by changes in the solution volume upon mixing. Mole fraction is also the concentration unit that has the strongest correlations, over the widest concentration ranges, with solution properties such as vapor pressure, osmotic pressure, and chemical potential.^73,74^ Generally, NaCl is modeled as a strong electrolyte that fully dissociates in water to form two molar equivalents of cations and anions, following conventions from electrolyte theory. Under this framework, 360 g of NaCl will dissolve in 1 L of water, forming a saturated solution with effective mole fractions of 0.091 for Na^+^, 0.091 for Cl^−^, and 0.818 for water.

In a non-ideal mixture, the activity of a species is normally computed as the product of the effective mole fraction and an activity coefficient. The latter can be obtained from activity coefficient models derived from electrolyte theories. Conventional electrolyte models use dilute conditions as a reference point (Debye–Hückel and Pitzer equations) and extend their applicability to high concentrations using binary and ternary interaction parameters. Vapor pressure studies of saturated NaCl indicate a water activity of 0.755 at 20 °C, corresponding to an NaCl activity of 0.245 activity, greater than its effective mole fraction of 0.182. Conventional electrolyte theory would resolve this difference with an activity coefficient of 1.39. An alternative, mechanistic approach is offered by the hydration theory of salts.

Hydration plays a key role in the solvation of ionic salts, where the energy released from the formation of ion–solvent interactions aids in the overcoming of the lattice energy of the ionic salt. Hydration of the ions also influences the colligative properties of concentrated mixtures,^74–77^ such as vapor pressure lowering, boiling point elevation, freezing point depression, and osmotic pressure. A hydrated ion is cloaked in the solvent molecule and thus, interactions between the hydrated shell and bulk water are very similar to the interactions between water molecules. Because of this cloak, the mixing of salts with water can be described surprisingly well by the ideal Raoult's law.^73^ Many salt solutions behave ideally when the ratio of the solute to the solvent is relatively large, once hydration is considered. The hydrated hydrodynamic radius (Stokes radius) is required to describe the diffusion rate of cations^78,79^ and viscosity of their solutions.^80^ The selectivity of reverse osmosis and nanofiltration membranes depend on electric charge-based effects as well as sieving effects that depend on the hydrodynamic radius.^81^ While there are notable examples that consider hydration,^75,82–87^ It is surprising that many electrolyte models do not more explicitly include hydration effects despite our awareness of its influence.

In this study, sodium ions are modeled as being hydrated by 3.9 moles of water molecules,^76^ in which the hydrating waters are removed from the mole fraction attributed to the bulk solvent. As illustrated by Robinson and Stokes,^88^ the significance of the hydration number of an ion extends beyond its coordination number to quantitatively define the ion as a “kinetic entity” (the solution state of an ion most appropriate to model). When the water–ion bonding in this “kinetic entity” is energetically significant, it can be argued that the ion removes a stochastically significant fraction of water from the bulk solution. Some studies have considered assigning hydration numbers to the cation or anion as a matter of sematic bookkeeping.^75^ However, experimental evidence suggests that water solvates cations much more strongly than anions.^76^ This phenomenon is attributed to charge density surrounding the oxygen atom in water, which has twice the charge of each hydrogen atom, leading to stronger interactions between cations and water. Therefore, to remain consistent with the known trends from recent studies, we assign the number of hydrating waters based on the distribution of the cations.^76^

Applying this simple hydration model for a saturated aqueous NaCl solution results in an effective mole fraction of 0.281 and 0.719 for Na^+^(3.9H_2_O)·Cl^−^ and bulk water respectively. This ionized and hydrated mole fraction of 0.281 is a good estimate for the experimentally observed activity of 0.245 for saturated NaCl as it is consistent with the effects of ion pairing.^74,89^ Ion pairing reduces the observed activity of NaCl, and likely reaches a maximum at saturation, causing the observed activity to be slightly lower than the “ideal” ionized and hydrated mole fraction. The ionized and hydrated mole fraction framework will be used for the subsequent ternary diagrams.

### 3.2. Ideal MOS-driven FP process

**Fig. 2:**
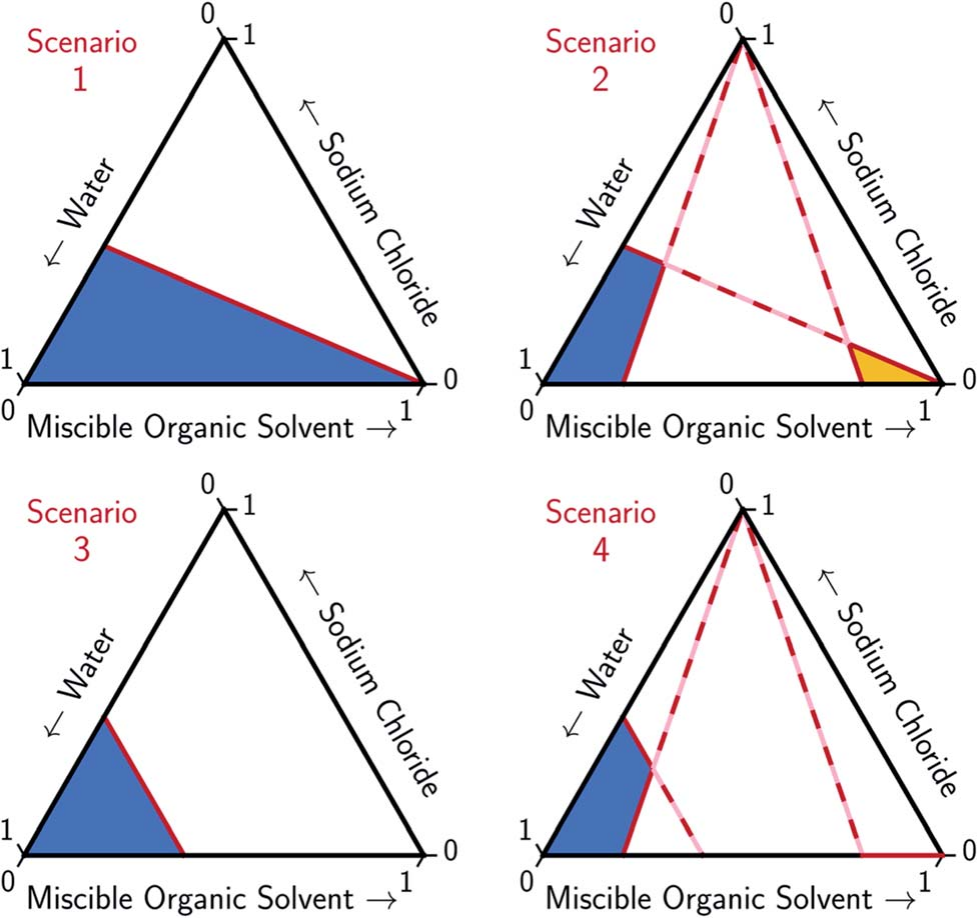
Limiting scenarios for MOS-driven FP expressed in ternary diagrams. Scenario 1 assumes that the solubility of the salt is proportional to the moles of water regardless of the presence of MOS, with no competition between the solute and MOS. Scenario 2 adds the assumption that the salt and MOS have limited solubility. The limiting concentration of both the salt and MOS are proportional to the moles of water (no solute competition). Scenario 3 depicts the solubility of a salt that is limited by the mole fraction of water competing with MOS. Scenario 4 adds that both the salt and MOS have limited solubility. The solubility of the salt is limited by either the mole fraction of water competing with solute MOS or the solubility of the MOS. The solubility of the MOS is proportional to the moles of water (no solute competition). Liquid aqueous- and organic-rich regions of each phase diagram are denoted by blue and yellow shading, respectively.

**Fig. 3:**
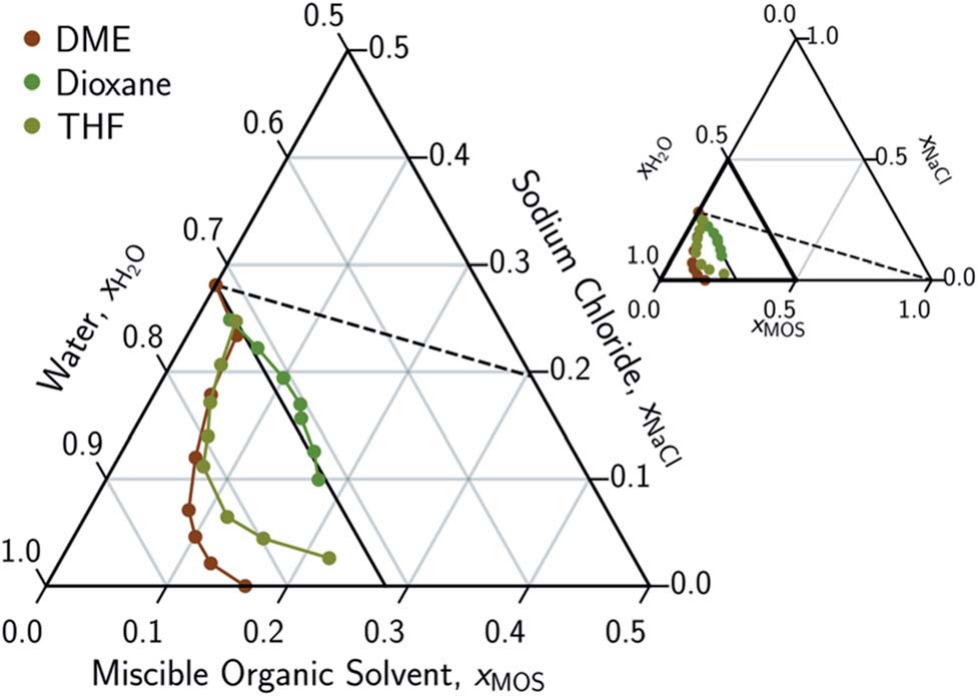
Ternary phase diagram focusing on the aqueous-rich phase of water–NaCl–MOS systems for ethers. Primary phase diagram focuses on the aqueous-rich quadrant (0.5 < *x*_H_2_O_ < 1.0, 0.0 < *x*_NaCl_ < 0.5, and 0.0 < *x*_MOS_ < 0.5) of the full phase diagram (inset). The concentration of the dimethyl ether (DME), tetrahydrofuran (THF), and dioxane are measured using NMR and plotted in terms of an ionized and hydrated salt (Na^+^(3.9H_2_O)·Cl^−^). Theoretical limits based on minimal MOS interaction, Scenario 1 (eqn (1), dashed black line), and solute displacement mechanism, Scenario 3 (eqn (2), solid black line). Phase diagrams were generated using Python-Ternary.^91^

**Fig. 4:**
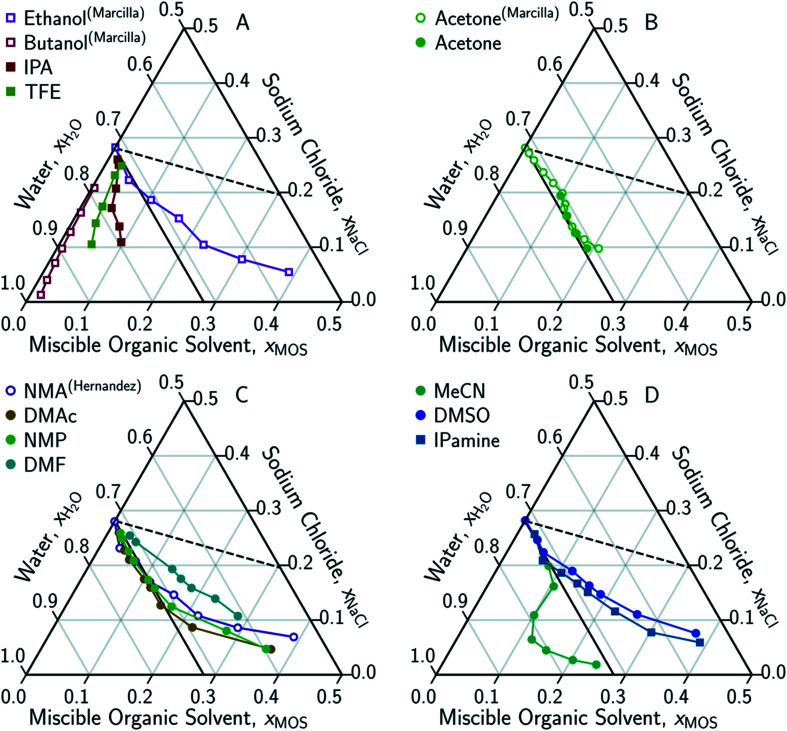
Ternary phase diagrams examining the aqueous-rich phase of water–NaCl–MOS for: (A) alcohols; (B) acetone; (C) amides; and (D) other polar solvents. Solid symbols denote data points measured in this work, while hollow symbols represent literature data from Marcilla *et al.*^20,21^ for ethanol, butanol and acetone and from Hernandez *et al.*^22^ for *N*-methylacetamide presented here for comparison and validation. Square and circular markers denote protic and aprotic solvent, respectively. All data points are plotted in terms of the ionized and hydrated salt (Na^+^·3.9H_2_O·Cl^−^). Theoretical limits based on minimal MOS interaction, Scenario 1 (eqn (1), dashed black line), and solute displacement mechanism, Scenario 3 (eqn (2), solid black line). Phase diagrams were generated using Python-Ternary.^91^

**Fig. 5:**
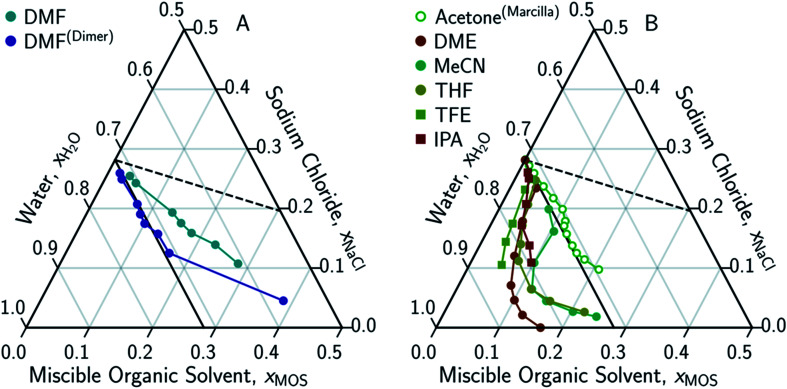
Ternary phase diagrams examining the aqueous-rich phase of water–NaCl–MOS for: (A) DMF when plotted monomolecularly and as a dimer; and (B) alcohols, acetone and MOS that exhibited a LL boundary in addition to a LS boundary. Phase composition was determined through NMR studies (or obtained from the literature) and plotted in terms of an ionized and hydrated salt (Na^+^·3.9H_2_O·Cl^−^). Theoretical limits based on minimal MOS interaction, Scenario 1 (eqn (1), dashed black line), and solute displacement mechanism, Scenario 3 (eqn (2), solid black line). Phase diagrams were generated using Python-Ternary.^91^

An ideal MOS is one which, when added to the brine in a minimal amount, causes the bulk precipitation of the dissolved salt. The full potential of MOS-driven FP has not been realized because the mechanism by which MOS displaces salt has yet to be determined. In this paper, models for two ideal solvation scenarios, as illustrated in Fig. 2, are compared to the experimental data and the associated trends found in Fig. 3–5. The first scenario models the MOS as a diluent that does not form energetic interactions with NaCl and water (Scenario 1). The second scenario models the energetic interactions between MOS and water to be identical to that between NaCl and water, while maintaining the assumption of zero interactions between MOS and NaCl (Scenario 3). These simplified scenarios provide a baseline for comparison between the different water–NaCl–MOS ternary systems to ascertain their viability for MOS-driven FP and other water treatment processes.

#### 3.2.1 Scenario 1 – minimal MOS interaction

In the first scenario, MOS is modeled as a diluent, based on the assumption of negligible MOS–NaCl and MOS–water energetic interactions. Thus, the solubility of NaCl in the ternary mixture is limited by the amount of water, which decreases proportionally with the addition of MOS.^60,90^ Starting from this assumption, the solid–liquid (SL) phase boundary in the ternary diagram can be estimated, using saturated aqueous NaCl as a reference point. This is modeled by eqn (1), where the relative hydrated mole fraction between the NaCl and water for any given concentration (*x*(Na^+^(3.9H_2_O)·Cl^−^) and *x*(H_2_O), respectively) is equivalent to that at the saturated point of NaCl in water (denoted by the subscript ‘Aq,Sat’).1a
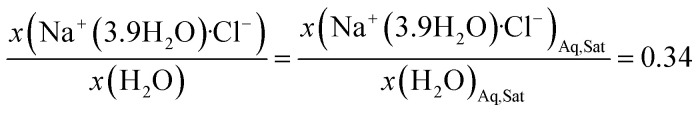

1b *x*(H_2_O) + *x*(Na^+^(3.9H_2_O)·Cl^−^) = 1 − *x*(MOS)

In conducting this study, an important aim was to learn how DME compared to other potential MOS. We included partially-miscible two liquid-phase systems (butanol and DME) and thirteen fully water-miscible MOS in Fig. 3 and 4. The fully water-miscible MOS could be further differentiated into (1) systems that remained as a single aqueous phase in the presence of NaCl (NMA, DMAc, NMP, IPamine, DMSO, and EtOH) and (2) systems that exhibit both aqueous and organic phases upon addition of NaCl (MeCN, THF, TFE, IPA, acetone). The two-phase systems added a liquid–liquid (LL) boundary that is distinct from the SL boundary. The existence of an LL boundary provides an added potential for water selective solvent extraction.^40–59^

To account for the phenomenon of liquid phase separation in the conceptual models, solubility limits were imposed for the aqueous phase in eqn (1) to describe a partially-miscible organic solvent, thereby superimposing a liquid–liquid phase equilibria boundary on Scenario 1, as depicted in Scenario 2 of Fig. 2. However, the modified model failed to conform to the curvatures in the LL boundary that is especially pronounced for DME, MeCN, and THF in Fig. 3 and from the literature.^20–22,60–66^

#### 3.2.2 Scenario 3 – solute displacement mechanism

Rather than assuming zero solute interactions between MOS and water, and between MOS and NaCl, it may be more useful to conceptualize the solubility of an ionizable salt from the thermodynamics of solvation, *via* Hess's Law. When a solute is dissolved, the bonds within the solid (lattice energy, hydrogen bonding, and other solid-state intermolecular bonding) are replaced with bonds to the solvent, *i.e.* solvation, which includes hydrogen bonding and a range of transient bonding phenomena that are common in the liquid state. For a salt to dissolve, the lattice energy must be overcome by the solvation energy. The saturation point of a mixture at a given temperature and pressure is in part dependent on the chemical potential of the solvent.

Under this framework, solutes will compete directly with each other for the common solvation agent of water (*x*_w(hydrated)_). Thus, the solubility of all solutes in the aqueous phase would be limited by the hydrated mole fraction of NaCl for saturated aqueous NaCl, *x*(Na^+^(3.9H_2_O)·Cl^−^)_Aq,Sat_. Under this limiting scenario, the addition of MOS (*x*(MOS)) to a saturated aqueous NaCl mixture will induce further precipitation of NaCl (*x*(Na^+^(3.9H_2_O)·Cl^−^)) by an equivalent molar quantity. This one-to-one molar substitution of dissolved solute is depicted as Scenario 3 in Fig. 2, and eqn (2). Scenario 3 effectively describes the LS phase boundary limit on water–NaCl–MOS mixtures involving NaCl at low MOS concentrations, as featured in Fig. 3 and 4, and in other reports.^20–22,60–66^

2a *x*(Na^+^(3.9H_2_O)·Cl^−^)_Aq,Sat_ = *x*(Na^+^(3.9H_2_O)·Cl^−^) + *x*(MOS)

2b *x*(H_2_O) + *x*(Na^+^(3.9H_2_O)·Cl^−^) + *x*(MOS) = 1

2c *x*(H_2_O)_Aq,Sat_ = *x*(H_2_O)

There are no scenarios in Fig. 2 representing how changes in the solution's dielectric constant would adjust the solubility of NaCl in a mixed solution. In general, the dielectric constant changes with the solvent's mass fraction,^92^ with the degree of change dependent on the specific MOS's properties. As a result, there is no way to easily plot a dielectric-based governing limit in mole ratio terms similar to those in Fig. 2. However, the inability to plot a model based on dielectric limitations does not appear to be a problem. At low MOS and high NaCl concentrations, all the data collected here match a one-to-one molar exchange of NaCl and MOS (with DMF as the only exception). As this is a molar trend, the results are inconsistent with processes governed by dielectric constants. If the process were governed by solution dielectric changes, then dioxane should drive a much steeper decline in NaCl concentration than MeCN (Fig. 3 and 4), when plotted in terms of mole ratio, based on the differences in dielectric constant and molecular mass. Dioxane has a dielectric constant of 2.2 (much lower than pure water's dielectric constant of 78.4 or concentrated aqueous NaCl solution's dielectric constant of <40),^93^ and a relatively high molecular mass of 88 g mol^−1^. MeCN has a higher dielectric constant of 36.6, and lower molecular mass of 42 g mol^−1^.^94^ The results featured in Fig. 3 and 4 indicate that dioxane, MeCN, and the rest of the MOS studied (except DMF) here are colinear with the predictions from the solute displacement model, at low MOS concentration and high NaCl concentration. Under these conditions, the systems appear to be well described by a mechanism in which the MOS solute and salt compete with each other for hydrating water molecules and can be used as a limiting case for subsequent FP studies involving nascent water–salt–MOS systems.

The only MOS to deviate from the one-to-one solute displacement is DMF. Previous experiments involving DMF and other formamides have displayed evidence of dimerization in chlorinated solvents.^95,96^ If the ternary data for DMF is plotted as dimer (Fig. 5A), the resultant points matches the predictions from the solute displacement model. This is consistent with our previous work in which we found evidence for similar aqueous dimers, which have been observed but are difficult to measure and identify.^97^

The one-to-one exchange of MOS for solute is in its most abstract a thermodynamically reversible process; and provides a clear basis for optimizing the efficiency of MOS driven FP. Given a one-to-one molar exchange, the mass of MOS required to precipitate a given mass of salt will vary in proportion to the molecular mass of the MOS. Systems using MOS would benefit from low molecular weight if the MOS must undergo phase changes (evaporation), given that the energy of phase changes, such as enthalpy of vaporization, correlate with the mass more closely than the molar quantity for similar materials.

### 3.3. Non-idealities of MOS-driven FP process

Scenario 3 (one-to-one displacement) does not capture all the aqueous phase behavior of the ternary water–NaCl–MOS system. Most MOS remove between 15–90 g kg^−1^ TDS of NaCl (0.25–3.1 molar MOS) before reaching the LL boundary, or, in the case of NMA, DMAc, NMP, IPamine, DMSO, and EtOH, display increasingly “non-ideal” behavior with a less than one-for-one molar exchange of MOS for NaCl. While this is a challenge for the bulk removal of highly soluble salts, like NaCl, the removal of 15–90 g kg^−1^ TDS would easily address most sparingly-soluble scalants. For instance, we demonstrated that 97.7% of the CaSO_4_ can be precipitated from a saturated CaSO_4_ solution through the addition of DME. Studying CaSO_4_ comes with its own challenges.

Under these conditions the displacement of CaSO_4_ by DME is not one-to-one, as a DME mole fraction of 0.167 displaces calcium sulfate from a solution with a CaSO_4_ mole fraction of 5.5 × 10^−4^. Due to the difficultly of quantitatively handling DME, it has not been possible to determine experimentally whether the displacement of CaSO_4_ is initially an ideal one-to-one displacement which becomes non-ideal as the DME concentration increases or if it is non-ideal the entire time. It is however possible to consider other salts. Based on the water–NaF–NMA system,^22^ salts that saturate at low salt mole fractions (<0.03) appear to deviate from one-to-one displacement phenomenon. Perhaps sparingly soluble salts are less sensitive to the water concentration which is in large excess at their saturation in contrast to highly soluble salts where the water concentration is on the same order of magnitude as the salt at saturation (especially when hydration is considered). This deviation from one-for-one molar precipitation is to be expected given that scalants remain in solution in the presence of modest concentrations of organics and other solutes. It has, however, been noted that for the same relative volume of MOS, a greater fraction of a modestly soluble solute will precipitate than a highly soluble electrolyte.^2,4,5,22,98^ Thus DME should be effective at precipitating scalants even if an excess is required.

The concentrations of the MOS and the salt appear to influence the range over which the one-to-one solute displacement mechanism is applicable. It remains to be determined whether the process is attenuated with concentration or if another mechanism governs precipitation at low concentrations. In the case of attenuation, the original solute displacement model (eqn (2)) can be modified by incorporating an exponentially decaying term, which defines the solute sensitivity to water (*ε*_is_). Furthermore, the competing effects arising from the self-solvation of the MOS can be incorporated as a fitting parameter (*ε*_jj_), to model the diminishing impact of MOS concentrations on the LS boundaries in Fig. 4, yielding eqn (3). In this form, the order of solute precipitation is maintained with low soluble solutes precipitating prior to highly soluble salts. As a result, FP and water selective extraction should allow for the selective removal of a salt from complex mixtures. Furthermore, a large fraction of low solubility salts can still be removed with an excess of another solute. A total of 97.7% of the CaSO_4_ can be precipitated from a saturated CaSO_4_ solution by increasing the DME concentration to 0.167 mole fraction, which is approximately 25% by mass.3
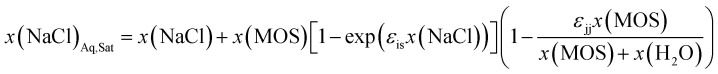


Eqn (3) is able to model nearly all the data that deviates positively from the solute displacement mechanism (Scenario 3, eqn (2)) in Fig. 4. The most significant positive deviations from Scenario 3 represent LS phase boundaries. Negative deviation from Scenario 3, Fig. 5B, represents a transition from LS to LL equilibria, which is not captured by eqn (3). Several of these LL phase boundaries are non-linear (DME, acetone, MeCN, and THF) as shown in Fig. 5B. For a few of the MOS (dioxanes and IPA), the dominant phase boundary appears to be closely balanced between a LL and a LS boundary, with the introduction of the MOS initially inducing LL separation followed by precipitation.

Interestingly, a NaCl-driven LL boundary was identified for wine by an alchemist in the 1200s.^2,99^ The water–NaCl–EtOH system only has a LS boundary, suggesting the LL boundary observed for wine requires additional solutes (sugars amongst other organics).

### 3.4. Implications on MOS selection for MOS-driven FP and MOS-based water selective extraction for bulk desalination

Removal of highly soluble salts (>90% TDS) by MOS can be achieved with (1) fractional precipitation through the addition of large amounts of MOS, or with (2) selective solvent extraction^40–59^ through the formation of a second, hygroscopic organic phase.

As deviation from Scenario 3 results in diminishing returns, a large excess of MOS is required to precipitate a highly soluble salt through FP. Furthermore, even if reasonably low concentrations of a highly soluble salt can be attained through FP, the removal of MOS from the waste stream must be considered. Single-phase MOS, including NMA, DMAc, DMF, NMP, IPamine, DMSO, and EtOH, have very low activity coefficients at infinite dilution, indicating a high water affinity.^100^ The problem is compounded as these single-phase MOSs are notoriously difficult to fully separate from water using conventional methods due to their ability to pass through membranes, low-vapor pressures, high-boiling points, and the formation of azeotropes with water. Therefore, FP is likely not well suited for total TDS removal in zero liquid discharge (ZLD) applications.

The introduction of an MOS-driven FP process that induces separation only near the saturation point of highly soluble salts could be incorporated into a ZLD crystallizer to protect the heat exchangers from fouling, and/or allow a more rapid introduction of heat. However, the inclusion of an MOS would likely complicate the crystallizer treatment train. Further analysis is required to determine if inclusion of MOS in crystallizers would provide an economic benefit.

On the other hand, there are distinct advantages in selecting solvent extraction for ZLD applications. In solvent extraction, MOSs that are capable of phase separating from water into two liquid phases, namely an organic-rich and a water-rich phase, are employed. These include natural two-phase MOSs, such as DME which phase separates naturally at higher MOS concentrations, and MOSs that only attain two liquid phases in the presence of salt (*e.g.* MeCN, THF, and acetone). When the MOS is carefully selected, the generation of the hygroscopic, organic-rich phase can selectively solvate water while retaining the salt ions in the aqueous-rich phase. Thereafter, the organic phase can be preferentially siphoned out and de-watered using traditional separation processes (*e.g.*, distillation columns). The product water can be optimized for total TDS removal or ZLD through process control optimization.

For both the native and salt-induced two-phase systems, designing selective solvent extraction by a hygroscopic organic phase will require concentration dependent water solubility of the organic phase. The relationship between concentration and chemical potential dictates the energy efficiency of water removal over various concentration ranges. This data can be supplied by the chemical potential of solutions or compositional tie lines from phase analysis. Currently this experimental data does not exist for the two-phase MOS studied in this report. Once this information is established, it will be possible to compare water selective solvent extraction with MOS to other high concentration dewatering and ZLD technologies.

## 4. Conclusion

Gross trends in the aqueous phase boundaries of ternary water–NaCl–MOS systems have been identified by studying a series of MOS with aqueous NaCl. These trends include a one-for-one molar exchanger of hydrated NaCl for MOS at dilute MOS and high saturated salt concentrations, that can be explained with the solute displacement mechanism. This relatively “ideal” behavior for saturated NaCl solutions corresponds to conditions that are clearly non-ideal from the perspective of traditional electrolyte models. The ability of the solute displacement mechanism to independently describe the system diminishes as MOS concentrations increase, presumably due to competing effects from self-solvation. The one-to-one exchange also does not hold for salts that saturate at low mole fraction (<0.03). It is undetermined whether the sensitivity of these salts is attenuated with concentration or if another mechanism governs precipitation at low concentrations. Despite diminished performance at low salt concentrations, 97.7% of the CaSO_4_ can be precipitated from a saturated CaSO_4_ solution by increasing the DME concentration to 0.167 mole fraction (∼25 wt%). This work has also identified several fully water miscible MOS (with relatively low water affinity) that develop a LL boundary as the MOS concentration is increased. The aqueous portion of these salt-induced two-liquid phases was quantified.

In terms of using DME for an MOS-driven FP process, these data have illuminated three points. First, increasing the overall fraction of a highly soluble salt removed *via* MOS-driven FP (*i.e.*, extending the LS boundary) offers only a limited advantage. Between diminishing returns for MOS addition and the difficulty of removing the solvents, extending the LS boundary is not a promising pathway to optimize MOS-driven FP. An MOS that natively forms two-phases with water also offers process design advantages. As a result, two-phase DME is well positioned for use in an MOS-driven FP process.

Second, MOS-driven FP has been demonstrated to remove a greater fraction of low soluble salt per unit MOS, even if a one-to-one removal is not achieved. Here, we have demonstrated that 97.7% of CaSO_4_ can be removed from a saturated solution with DME. This separation is still believed to be achieved with a molar mechanism, so DME, which consists of three heavy atoms, will minimize the volume of MOS required to achieve the scalant removal. Most scalants have a lower solubility than CaSO_4_; and, based on trends, a greater fraction should be removed for the same quantity of DME. Based on the proposed mechanism, MOS-driven FP should function well regardless of the solution's concentration and possibly better for a mixed solution with high overall TDS concentrations. DME-driven FP may be a general method to efficiently remove the vast majority of scalants (and may also function as a biocide) in SWRO and other processes, regardless of the solution's overall concentration, minimizing the stoichiometric use of anti-scalants, which leave residual chemicals in the brine. Such a softening technology does not currently exist.

Third, optimizing the removal of MOS from the product water (and concentrate byproducts) *via* an MOS with a low boiling point, is likely the most important feature for developing and deploying MOS-driven FP. As a condensable gas, DME readily separates from any solution or solid, rendering DME uniquely positioned to drive MOS-driven FP processes and effectively recycled.

## Conflicts of interest

There are no conflicts to declare.

## Supplementary Material
